# Variability of Bioactive Glucosinolates, Isothiocyanates and Enzyme Patterns in Horseradish Hairy Root Cultures Initiated from Different Organs

**DOI:** 10.3390/molecules24152828

**Published:** 2019-08-02

**Authors:** Regina Bertóti, Andrea Böszörményi, Ágnes Alberti, Szabolcs Béni, Márta M-Hamvas, Éva Szőke, Gábor Vasas, Sándor Gonda

**Affiliations:** 1 Department of Pharmacognosy, Semmelweis University, Üllői út 26, H-1085 Budapest, Hungary; 2 Department of Botany, Division of Pharmacognosy, University of Debrecen, Egyetem tér 1, H-4010 Debrecen, Hungary

**Keywords:** hairy root, horseradish, glucosinolate, isothiocyanate, nitrile, myrosinase, horseradish peroxidase

## Abstract

Horseradish hairy root cultures are suitable plant tissue organs to study the glucosinolate–myrosinase–isothiocyanate system and also to produce the biologically active isothiocyanates and horseradish peroxidase, widely used in molecular biology. Fifty hairy root clones were isolated after *Agrobacterium rhizogenes* infection of surface sterilized *Armoracia rusticana* petioles and leaf blades, from which 21 were viable after antibiotic treatment. Biomass properties (e.g., dry weight %, daily growth index), glucosinolate content (analyzed by liquid chromatography-electronspray ionization-mass spectrometry (LC-ESI-MS/MS)), isothiocyanate and nitrile content (analyzed by gas chromatography-mass spectrometry (GC-MS)), myrosinase (on-gel detection) and horseradish peroxidase enzyme patterns (on-gel detection and spectrophotometry), and morphological features were examined with multi-variable statistical analysis. In addition to the several positive and negative correlations, the most outstanding phenomenon was many parameters of the hairy root clones showed dependence on the organ of origin. Among others, the daily growth index, sinigrin, glucobrassicin, 3-phenylpropionitrile, indole-3-acetonitrile and horseradish peroxidase values showed significantly higher levels in horseradish hairy root cultures initiated from leaf blades.

## 1. Introduction

Horseradish (*Armoracia rusticana* P. Gaertner, B. Meyer & Scherbius) is a Brassicaceae plant, which is native to southeastern Europe and western Asia. The principal commercial horseradish producing countries are the United States and Hungary [1]. Horseradish is used today primarily as a condiment, however has also been known as a medicinal herb since antiquity [1,2,3]. 

**Figure 1:**
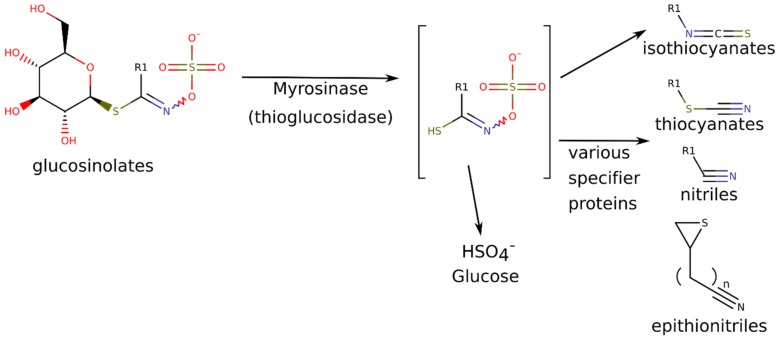
Conversion of glucosinolates into isothiocyanates and other various volatile breakdown products, depending on reaction conditions. Characteristic glucosinolates and breakdown products in horseradish include: R^1^ = allyl (glucosinolate: sinigrin, specific decomposition products: allyl isothiocyanate, allyl thiocyanate and allyl nitrile); R^1^ = 2-phenylethyl- (= phenethyl-) (glucosinolate gluconasturtiin, specific decomposition products: 2-phenylethyl-isothiocyanate, 2-phenylethyl-thiocyanate and 3-phenylpropionitrile) [2,3,4,5].

For both utilizations, the pungent, lacrimatory compounds, the isothiocyanates (ITCs) are responsible. The isothiocyanates are the default hydrolytic breakdown products of the glucosinolates (GLS). Glucosinolates are N-hydroxy-sulfates with a highly variable side chain (R) and a sulfur-linked beta-d-glucopyranose (Figure 1). GLSs are odorless molecules found in vacuoles, while the myrosinase enzyme (MYR), which catalyzes the hydrolytic reaction, is stored in different compartments, typically in myrosin cells [2].

Glucosinolates (GLSs) are the precursor molecules of the biologically active ITC components. Seventeen GLSs, including glucoiberin, sinigrin (SIN), 2-methylsulfonyl-oxo-ethyl-GLS, gluconapin, glucocochlearin, glucoconringianin, glucosativin, glucoibarin (GIB), 4-hydroxyglucobrassicin, neoglucobrassicin (NEO), glucocapparilinearisin or glucobrassicanapin, glucotropaeolin, glucobrassicin (BRASS), gluconasturtiin (GLN), 4-methoxyglucobrassicin, glucoarabishirsutain (ARAB) have been detected in horseradish so far [2].

Isothiocyanates (ITCs) are volatile compounds, consisting of an isothiocyanate group (–NCS) and an R side chain, same as that of the corresponding GLS, which influences, among others, the bioactivity.

The key constituents of horseradish root essential oil are allyl isothiocyanate (AITC, 44.3–81.8%) and 2-phenylethyl isothiocyanate (PEITC, 4.2–51.3%) [3,4,5,6,7]. The following minor ITCs have been also described in horseradish root: isobutyl isothiocyanate, 4-isothiocyanato-1-butene, butyl isothiocyanate, 3-methylbutyl isothiocyanate, pentyl isothiocyanate, 4-methylpentyl isothiocyanate, benzyl isothiocyanate [2], 7-methylsulphinylheptyl isothiocyanate, 6-methylsulphinylhexyl isothiocyanate, 5-methylsulphinylpentyl isothiocyanate, 4- pentenyl isothiocyanate, 3-butenyl isothiocyanate and n-butyl isothiocyanate [3].

ITCs, especially AITC and PEITC, have several biological and possibly medicinal effects. As recently reviewed in [2,3], ITCs have strong anticarcinogenic and antimicrobial effects. AITC, PEITC and butyl ITC were proven to be anticarcinogenic e.g., on lung, prostate and bladder cells in animal models. The mechanism of action is mainly through inhibition of phase I (CYP) enzymes, as well as increasing the gene expression of phase II enzyme (e.g., GST), or, epigenetic regulation through miRNAs [8]. ITCs exert antimicrobial effects both on Gram-positive and Gram-negative bacteria, on yeasts and molds [9]. ITCs also have anti-platelet, gastro-protecting, plasma cholesterol lowering, and insecticidal activities [2].

Nitriles also carry the side chain from their precursor glucosinolate, as the side-product of myrosinase (MYR) hydrolysis, elemental sulphur is released. Nitriles are also usually volatile components [8]. Nitriles with an indole side chain, e.g., indol-3-acetonitrile have anticarcinogenic and insecticidal activities [9,10].

Horseradish myrosinase (MYR, beta-thioglucoside glucohydrolase) is a 65 kDa weight S-glucosidase enzyme consisting of two similar subunits linked by a zinc atom [3,11]. Myrosinase is not a substrate specific enzyme, it can catalyze hydrolysis of variable GLSs [11]. At least three MYR isoenzymes have been described (MyrA, MyrB, MyrC), their presence was species- and organ-specific [8,12,13]. Another MYR classification is based on tissue specific expression pattern: MYRI is specific to above ground organs, including the MyrA, MyrB, MyrC and AtTGG1-3 isoenzymes; MYRII is characteristic to roots, including AtTGG4 -5 and others [14]. When the plant tissues are damaged (e.g., by crushing), the myrosinase comes in contact with the GLSs, resulting in the release of bioactive ITCs, nitriles, thiocyanates, epithionitriles, or oxazolidines, depending on the reaction conditions, the substrate, and the presence/absence of specifier proteins [1,2,3,14]. ITCs are the main products of this reaction at a pH range of 5–8 and at 20–45 °C [3,15]. 

Horseradish has also been researched for its abundant enzyme horseradish peroxidase (HRP). HRP is a 44 kDa protein, consisting of a single polypeptide chain composed of 308 amino acids, a hemin prosthetic group and two calcium ions, which take part in the maintenance of enzyme conformation [16,17]. HRP indicates a set of specific enzymes like glutathione peroxidase or nicotinamide adenine dinucleotide (NADH) peroxidase [16]. It also takes part in the plant’s defense system through lignification and in recovery mechanisms, e.g., infected or damaged plant tissue, too [16]. This enzyme is used in several molecular biology methods: measuring blood glucose and cholesterol levels; estimating the content of other enzymes; analysis of immunoassays (e.g., enzyme-linked immunosorbent assay (ELISA), Western blot), or the purification of DNA probes [1,3,16]. It is also used in the meat industry [18].

Hairy root cultures are initiated by the infection of plant tissues by *Agrobacterium rhizogenes*. The transfer-DNA (T-DNA) of the bacterial Ri (root inducing) plasmid, containing the virulence genes *RolA*, *RolB* and *RolC*, integrates into the plant genome, causing hairy root disease [19,20]. The integration of these virulence genes can also result in changes in the secondary metabolite synthesis [20]. The neoplastic hairy root clones (HRCs) are capable of unlimited growth, are genetically stable in hormone free media (some could be maintained for 16 years [21]). They are suitable for production of high levels of secondary metabolites (e.g., pharmaceuticals, flavors, pigments) [20,22,23,24]. As a consequence of the gene transformation, secondary metabolite biosynthesis differs from that in the native plants [20,23]. Indole GLS levels showed higher values in hairy roots of *Brassica rapa*, *Sinapis alba* and *Eruca sativa*, than in the mother plant, but the total GLS content was lower in each case in the HRCs [25,26]. Hairy root cultures also tolerate scale-up and breeding in bioreactors well [23]. Moreover, hairy root cultures are also suitable subjects of functional gene analysis [27]. Horseradish does not develop fertile seeds, but Nakashimada et al. [28] and Repunte et al. [29] could establish artificial seeds from horseradish HRCs. Horseradish HRCs are also suitable for phytoremediation, or for sewage treatment [28,29]. 

Horseradish HRCs have been successfully used for peroxidase production several times [30,31,32,33,34,35]. The gene-transformed HRCs not only show high biomass production [18], but their peroxidase activity was also increased; the increase can be 20-fold [33]. Further, elicitation with *Verticillum* sp., plant extracts or heavy metals increased the HRP activity of horseradish HRCs [32,33,34,35,36,37]. For cultivation of horseradish HRCs, only air-lift bioreactors with polyurethane immobilization was suitable [38].

Horseradish roots are an extremely rich source of GLSs among Brassicaceae plants [2,37]. The secondary metabolite GLSs (precursors of bioactive ITCs) are produced in lower levels in horseradish HRCs, compared to the roots of the intact plant. Information is lacking on the glucosinolate–myrosinase–isothiocyanate system in hairy root cultures of horseradish. Although, in the Brassicaceae family, there are some example studies about GLS elicitation in HRCs. Alnsour et al. [39] used elicitation with increased MgSO_4_ in the media for GLS production. Wielanek et al. [40,41] successfully elicited GLSs in other Brassicaceae HRCs by feeding them precursor amino acids (phenylalanin, cystein). In *Brassica oleracea*, HRC GLS biosynthesis was influenced by the growth medium, and by the auxin concentration [42]. Chung et al. [43] raised GLS and phenoloid compound production and gene expression with copper oxide nanoparticles in *Brassica rapa* HRCs. Kinetin, a cytokinin, also increased the GLS content of *Brassica rapa* HRCs [25]. In *Eruca sativa* HRCs, aliphatic and indole GLSs were elicited with sulphur, but the treatment with ethephon and jasmonic acid increased the level of indole GLSs only [26]. Jasmonic acid also raised the level of indole GLSs in *Sinapis alba* and in *Brassica rapa* [25]. Kastell et al. [26] mentioned that compared to the mother plant, HRCs contained fewer GLSs, but more indole GLSs. In *Arabidopsis thaliana*, cytochrome P450 gene transformation resulted in a seven-fold rise of the aliphatic GLS content in third generation compared to wild HRC, but this GLS level was still very low compared to the mother plant [44]. 

The aim of the current study was to establish a set of horseradish HRC lines and study their chemical and enzymatic variability. The HRC lines were assayed for GLSs, myrosinase, and volatile decomposition products of GLSs, as well as for peroxidase. What is more, lines of leaf blade and petiole origins were compared for the above features. 

## 2. Results

### 2.1. In Vitro Horseradish Cultures

From the 50 isolated HRC lines, 21 were found to be viable; 10 from the inoculation of petiole (ArP) and 11 from the leaf blade (ArLB). All of the viable HRC lines have been created with the infection of *A. rhizogenes* A4. Strains T37, 15837 and 8196 of *A. rhizogenes* resulted in clones that became unstable during antibiotic treatment.

Presence of the bacterial *RolC* gene was confirmed by PCR analysis in each clone, using the DNA of *A. rhizogenes* A4 as a positive, and the DNA of mother plant as negative controls (Figure S1).

### 2.2. Identification of Glucosinolates in Horseradish Hairy Roots by LC-ESI-MS/MS Analysis

Based on their characteristic fragments [2,45,46,47,48,49,50] (Figure S2), several GLSs were identified (Figure S3) from the water extract of HRCs, listed in Table S1. The detected GLSs belong to several classes based on the side-chain, and include aliphatic, aromatic, indole-, and thiomethylalkyl ones as well. 

### 2.3. Detection of Nitriles and Isothiocyanates as GLS Breakdown Products from Horseradish Hairy Roots by gas Chromatography-Mass Spectrometry (GC-MS)

PEITC, the myrosinase breakdown product of (GLN) (Table S2, Figure S4) was found to be the main component of horseradish root culture ITCs. Not every abundant GLS was represented as an intensively corresponding ITC in the GC-MS samples. For example, aromatic ITC/nitrile compounds were not dominant, but 3-(methylthio)propyl isothiocyanate (MeSPITC), which is the breakdown product of glucoiberverin (IBER), was present in high amounts. In the background of this phenomenon, the differing MYR enzyme pattern of the HRCs can be expected, as detailed later.

Although seven GLSs were detected, from their breakdown products only three ITCs (AITC, MeSPITC, PEITC), and two nitriles could be identified (Table 1), from which 3-phenylpropionitrile (PECN) is the nitrile hydrolysis product of the main GLS, GLN [43,45]. 

### 2.4. Enzyme Content of Horseradish Hairy Roots

#### 2.4.1. Myrosinase Activity

**Figure 2:**
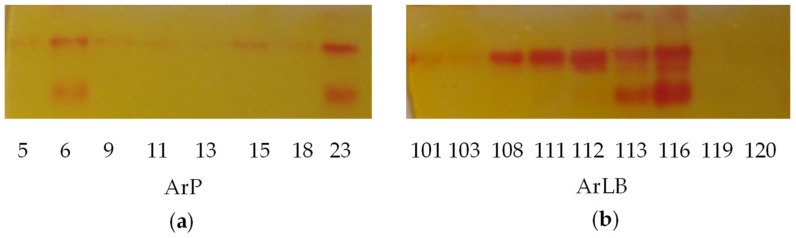
On-gel detection of myrosinase of the horseradish (*Armoracia rusticana*) hairy root clones, (**a**) initiated from petiole (ArP), (**b**) initiated from leaf blade (ArLB) shows visible variability. MyrB2 isoenzyme is present almost in all clones. Some hairy root clone contained three isoenzymes, which are ArLB113, ArLB116, ArP23. The detection solution contains methyl red which gives intensive red bands where the HSO_4_^2−^ is enzymatically released from the glucosinolates.

On native PAGE gels, three various MYR isoenzymes were observed (Figure 2a,b). The different pattern of the isoenzymes could account for the various patterns in volatile bioactive compounds. 

#### 2.4.2. Peroxidase Content and Activity

According to the literature [17,32], HRCs produce horseradish peroxidase. The two tested methods (gel analysis, and spectrophotometric assay) gave comparable results with both substrates (pyrogallol, guaiacol), correlation values were above 0.70 (*p* < 0.001). On the gels, five isoenzymes were visible (Figure S5). In the statistical analysis, total peroxidase activities of all isoenzymes were used.

### 2.5. Morphological Evaluations

Branching, adventitious shoot formation, and pink color of water extracts for GLS measurement were visually scaled, and used for the multi statistical analysis (Table S3, Figure S6).

### 2.6. Agrobacterial Inoculation of Different Horseradish Plant Organs Results in Different Feature Patterns in HRCs

Multivariate statistical analysis revealed interesting positive and negative correlations among the measured features, which included natural product concentrations, enzymatic activities and biological data such as growth value, dry weight, among others. These are detailed in the following.

**Figure 3:**
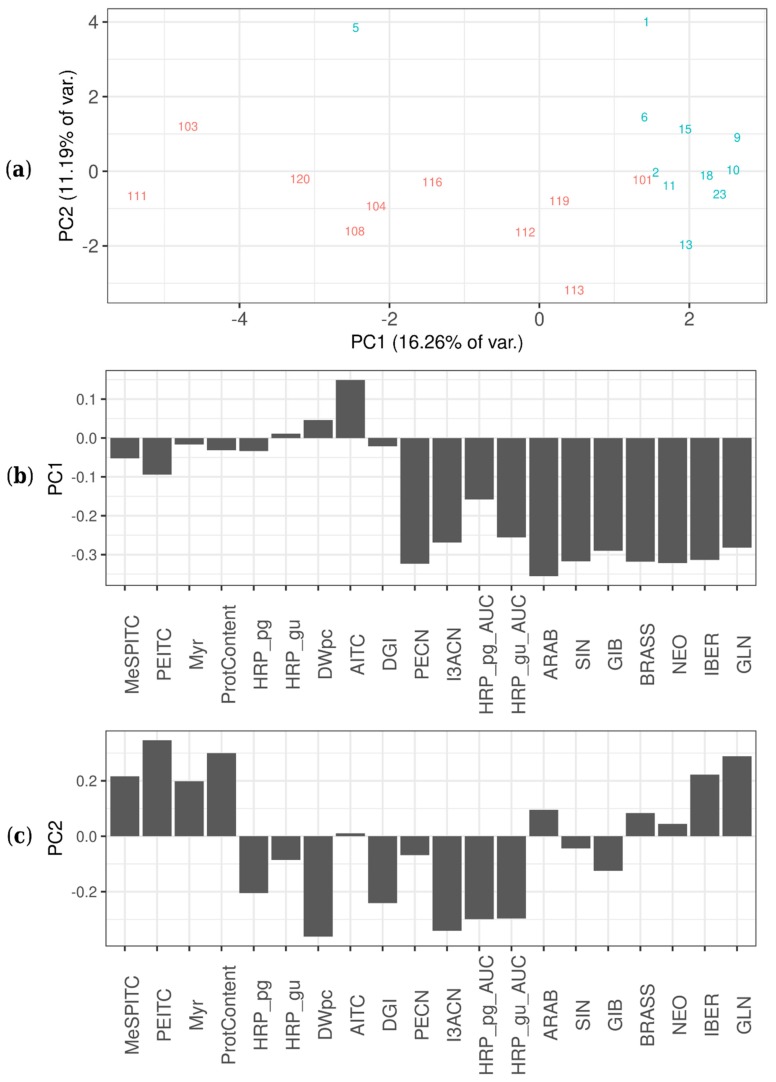
Principal component analysis score plot of all variables of the tested different horseradish (*Armoracia rusticana*) hairy root clones, including glucosinolate content, isothiocyanate content as well as enzyme activities/patterns. (**a**) Principal component analysis score plots showing different hairy root clones (HRCs) in the PC1—PC2 plane, separated by their features. The lines of different origins are shown with different colors: red—petiole, blue—leaf blade. (**b**) Contribution of features (loading values) to score values of horseradish hairy root cultures, in principle component 1 (PC1). Feature abbreviations: HRP_pg—horseradish peroxidase activity with pyrogallol as substrate (mmol pyrogallol min^−1^ mg^−1^ protein); HRP_gu—horseradish peroxidase activity with guaiacol as substrate (mmol guaiacol min^−1^ mg^−1^ protein); Myr—myrosinase activity; ProtContent—protein content (mg protein mg^−1^ fresh weight); MeSPITC—3-(methylthio)propyl isothiocyanate (abundance); PEITC—2-phenylethyl isothiocyanate (µg mg^−1^ DW); AITC—allyl isothiocyanate (µg mg^−1^ DW); DWpc—dry weight %; DGI—daily growth index; PECN—3-phenylpropionitrile (abundance); I3ACN—indole-3-acetonitrile (abundance); HRP_pg_AUC—horseradish peroxidase content with pyrogallol as substrate (abundance); HRP_gu_AUC—horseradish peroxidase content with guaiacol as substrate (abundance); ARAB—glucoarabishirsutain (abundance); SIN—sinigrin (µg mg^−1^ DW); GIB—glucoibarin (abundance); BRASS—glucobrassicin (abundance); NEO—neoglucobrassicin (abundance); IBER—glucoiberverin (abundance); GLN—gluconasturtiin (µg mg^−1^ DW). (**c**) Contribution of features (loading values) to score values of horseradish hairy root cultures, in principle component 2 (PC2). Feature abbreviations: same as in Figure 3b.

**Figure 4:**
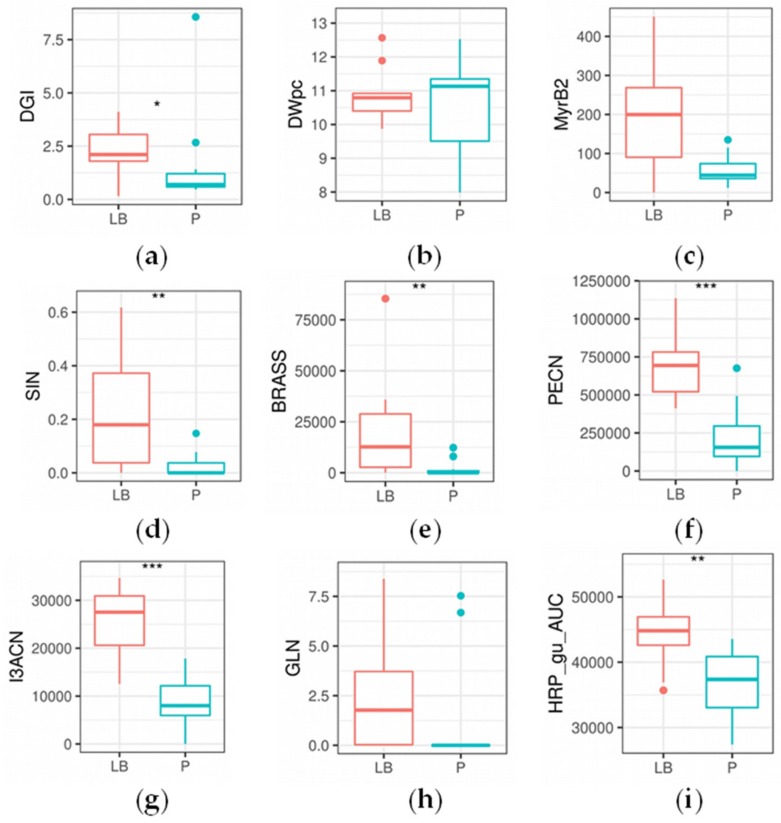
Boxplots showing statistical differences in glucosinolate and breakdown product concentration, enzymatic activity and general parameters between the horseradish hairy root culture lines of petiole (P) and leaf blade (LB) origin (*n* = 11 and *n* = 10, respectively). Significant changes are marked as follows: * *p* < 0.05; ** *p* < 0.01; *** *p* < 0.001; Kruskal–Wallis test. Subplots (**a**) daily growth index; (**b**) dry weight (%); (**c**) Myrosinase, activity of the second band as measured by on-gel analysis; (**d**) sinigrin content (µg mg^−1^); (**e**) glucobrassicin content (abundance); (**f**) 3-phenylpropionitrile content (abundance); (**g**) indole-3-acetonitrile content (abundance); (**h**) gluconasturtiin content (µg mg^−1^); (**i**) horseradish peroxidase content activity, as measured on-gel analysis with guaiacol as substrate.

Several significant differences were found between ArP and ArLB, groups according to the inoculated plant organ origin (ArP: petiole; ArLB: leaf blade) (Figure 3, Table 2). In all significantly different properties, ArLB was shown to have higher values. DGI was higher in ArLB lines (Figure 4a, *p* = 0.04864), while other growth parameters (e.g., DWpc) were not significantly different between the two groups (Figure 4b, *p* = 0.83269). From the enzymes, MyrB2 isoenzyme activity was higher, but not significantly in ArLB clones (Figure 4c; *p* = 0.08308). From the GLSs, all aliphatic (SIN, Figure 4d, *p* = 0.00730; IBER, *p* = 0.03864; GIB, *p* = 0.00278; ARAB, *p* = 0.03068), and two aromatic (BRASS, Figure 4e, *p* = 0.00291; NEO, *p* = 0.00153) GLSs were most abundant in ArLB HRCs (Table 2. Regarding the GLS breakdown products, only two nitriles showed significant differences between ArP and ArLB (PECN, Figure 4f, *p* = 0.00072 and I3ACN; Figure 4g, *p* = 0.00025), which have GLN and BRASS as precursors, respectively. Interestingly, the substrate GLN was not significantly different between the two groups (Figure 4h, *p* = 0.09426). The on-gel HRP activity in ArLB clones was significantly higher (Figure 4i, *p* = 0.00745).

As demonstrated in Figure 3a, the HRCs are separated into two groups according to the inoculated plant organ origin (ArP: petiole; ArLB: leaf blade), both groups show loose clusters with some compact subgroups. The separation of the two groups differing in the original organ (petiole versus leaf blade) is clearly visible along the PC1 dimension (Figure 3a). It can be recognized from the loading plot of PC1 (Figure 3b) that the difference between the two groups is mostly based on differences in the abundance of various GLSs (GIB, NEO, SIN, BRASS, ARAB, IBER and GLN), as well as peroxidase activity, and not because of biomass parameters (e.g., dry weight content (DWpc), protein content (ProtCont)). While the members of the ArP group contained 0.0272 ± 0.0710 µg mg^−1^ SIN, in the case of ArLB, the concentration was an order of magnitude higher, 0.2194 ± 0.3015 µg mg^−1^ (*p* < 0.01). The GLN content of ArP and ArLB HRCs was 1.2926 ± 4.2789 µg mg^−1^ and 2.4240 ± 4.0377 µg mg^−1^, respectively, but this difference was not statistically significant (*p* = 0.3760). Clones of ArP contained 10.4680 ± 1.4885% DW, clones of ArLB 10.8612 ± 0.8037% DW (*p* = 0.4818). Within-group variations (PC2 in Figure 3a) in both subgroups are mainly the result of variances in the DW%, protein content and, interestingly, total myrosinase activity.

On the heat map (Figure S7), the growing versus secondary metabolite production can be demonstrated through the negative correlation between several compounds (NEO, BRASS, IBER, GLN, ARAB, I3ACN) synthesis and biomass production (DGI, DWpc). In ArP clones GLSs (GLN, BRASS, SIN) are also anti-correlating with HRP activity (HRP_pg, HRP_pg_AUC). This is a phenomenon that is likely the result of the limitation of a rate-limiting nutrient; household processes and growth competes with defensive metabolite biosynthesis for resources. As glucosinolate biosynthesis not only requires carbon, but also S and N, this phenomenon is likely more expressed than in the case of other secondary metabolites.

## 3. Discussion

### 3.1. Comparison of Hairy Root and Native Root Glucosinolate Patterns

While SIN is the dominant GLS compound in native horseradish roots [2,3], the major GLS in HRCs was found to be GLN along with other aromatic/indolic GLSs: ARAB, BRASS. These are also present as minor components in native roots. GLN is usually present at about an order of magnitude lower than SIN in native roots [2,38]. Therefore, dominance of the aromatic GLS seems to be specific to HRCs.

Although the total GLS level of HRCs seems to be lower than in the mother plant, the indole GLSs were observed to be more dominant in HRCs compared to the mother plant [25,26,41,54,55].

In *Brassica rapa* ssp. *Pekinensis*, seven different MYB (myrosinase B) transcription factors were detected [56], which showed organ and GLS-class specific presence. MYB28 and MYB29 transcription factors are taking part in aliphatic GLS transformation, the level of which was different in the stem compared to other organs [56]. The tissue specific accumulation of GLSs and the transcription pattern in whole plants is distinct from that in in vitro cultures [44,54] In *Arabidopsis thaliana*, the level of transcription factors of aliphatic GLS genes was significantly lower in HRCs than in the native root [54]. 

Despite the low SIN-AITC content, the characteristic GLS-ITC/nitrile pattern of the mother plant *Armoracia rusticana* can be recognized (Table 1, Tables S1 and S2). The pattern is unlike that of *Armoracia macrocarpa,* because of the presence of IBER-MeSPITC, GIB, BRASS and ARAB [2,57]. Furthermore, the typical compounds of *A. macrocarpa*, 5-methylthiopentyl isothiocyanate (berteroin) and 6-methylthiohexyl isothiocyanate (lesquerellin) [57] are also missing from our horseradish HRCs. 

### 3.2. Indolic Glucosinolates and Their Breakdown Products

The connection between the indole GLS BRASS and indole-3-acetic acid (IAA) is worth a closer look. Firstly, the plant-derived indole GLSs could hydrolyze into I3ACN. This compound was shown to defend the plant against fungi [53]. However, I3ACN is also an intermediate in the biosynthesis of IAA [47,48]. During agrobacterial infection, T-DNA—which includes genes for auxin production (e.g., IAA)—is integrated into the plant genome [19], which causes the unlimited growth of the HRCs [58,59]. Thus, the detected I3ACN could derive from GLS hydrolysis as well as from the gene products of *Agrobacterium* [51,58,59,60]. Regardless of its source, the plant can use this component as an antifungal component, or as an intermediate for auxin (IAA) production to facilitate growth [8].

### 3.3. Agrobacterial Inoculation of Different Horseradish Plant Organs Results in Different Feature Pattern in HRCs

To our best knowledge, this is the first comparative study in which organ-dependent inoculation with *Agrobacterium* resulted in such differences in the Brassicaceae family, covering metabolites and enzymes, as well. A study has shown that GLS accumulation and transcription is tissue-specific, and differs from that in in vitro culture systems, like HRCs, in *Arabidopsis thaliana* [54], but no data on GLS content and enzyme activities was presented about HRCs initiated from different organs. Only in the case of *Coffea arabica* hairy roots was this phenomenon noted; inoculation of different embryotic organs resulted in different gene-expression patterns [61]. This could be partly because of the original hairy root induction protocols target leaves because of the consistency of the tissue [62]. The leaf-disc method (immersion of excised explant/leaf pieces into bacterial suspension) is the most widespread technique for initiation [35,36,63,64,65]. However, Saitou et al. [34,66] had infected both horseradish axial leaf midribs and petioles directly with a needle, and subsequently analyzed all of the created HRCs for peroxidase content. As in the present study, the HRP level was much higher in the HRCs, than in the leaves of in vitro plantlets. Unfortunately, in the paper of Saitou et al. [34], no data were presented regarding bioactive constituents. In another study, *Brassica rapa* hypocotyls, leaves and roots were inoculated with a needle. HRCs from the roots were not viable, and of the viable HRCs, 89% were isolated from the leaves, and only 11% from the hypocotyl [43]. The usually applied leaf seems to be a viable source of HRCs, as compared to the petiole.

### 3.4. Likely Dependence of Biologically Active Compound Concentrations on Myrosinase Isoenzyme Pattern

It is likely that the different MYR isoenzyme activities (Figure 2) are in part responsible for the significantly different aromatic nitrile concentrations between the ArP and ArLB groups. This is best presented by the following two, opposing phenomena: a significant positive correlation can be detected between abundance of a single Myr band’s intensity (MyrB2) and PECN (correlation value = 0.58, *p* = 0.014, Figure S8a); on the other hand, higher amounts of the same enzyme results in lower amounts of MeSPITC (correlation value = −0.516, *p* = 0.03, Figure S8b). Hence, decomposition by different enzymes likely influence the resulting pattern in HRCs. The effect does not have to be direct: lack of degradation by a myrosinase isoenzyme can also result in increased nitrile production after spontaneous decomposition, or decomposition by other enzymes or enzyme complexes, e.g., NSP, ESP [5,14,52,67,68,69].

Though not statistically significant, the organ of origin seems to influence the myrosinase pattern of the HRCs (Figure 2, Figure 4c). Organ-specific MYR activity and gene expression has already been described in Brassicaceae plants. Li and Kushad [70] observed that the leaves and roots of native horseradish plants have different MYR activity, regardless of the GLS content. Wittstock et al. [67] found that MYR isoenzymes and nitrile specifier proteins (NSP) are present in an organ-specific combinations in *Arabidopsis thaliana*. Although MYR is not a substrate-specific enzyme [11], through the different MYR binding proteins and MYR associated proteins, e.g., NSP or epithiospecifier proteins (ESP), enzymatic hydrolysis can result in different products e.g ITCs, nitriles [68].

In concordance with the above, our results showed an ITC–nitrile shift as a likely consequence of the changes in the abundance of a MYR isoenzyme [68]. Eriksson et al. [71] reported on tissue-specific expression of MYR gene families. Gene expression of MyrA isoenzymes were observed only in seed tissues, while MyrB isoforms were expressed in cotyledons and leaves. Furthermore, organ-specific expression of unique MyrB genes was also suggested, because seeds and leaves contained individual MyrB transcripts. Moreover, *Arabidopsis thaliana* also showed organ-specific NSP gene expression regulation [72]. Our results show that the enzyme pattern can significantly vary, and seriously influence volatile constituent patterns, not just overall yields. Also, these enzyme patterns seem to be influenced by the organ of origin in hairy root cultures.

Altogether, it is likely that the inoculation of different organs different enzyme expression patterns, which also results in differences in the GLS–MYR–ITC/nitrile system, results in the biosynthesis of different compounds.

## 4. Materials and Methods

### 4.1. Plant Material, In Vitro Cultures, Plant Transformation.

Roots of *Armoracia rusticana* (Gaertn. Mey. et Scherb.) were obtained from Királd, Hungary. The cleaned roots were immersed to fungasol, and planted to sterile perlite, and shoots were grown under plastic bottles. Newly appeared leaf blades and petioles of the roots were surface-sterilized by immersion in a commercial sodium hypochlorite solution for 30 min, three-fold diluted with water, containing 0.01% (*v*/*v*) Tween-80, under agitation. They were rinsed three times with sterile distilled water. The leaf blades and petioles were planted onto Murashige-Skoog [73] media with the addition of 3.0% (*w*/*v*) sucrose and solidified with 0.7% agar. Then, the surface-sterilized leaf blades and petioles were inoculated with *Agrobacterium rhizogenes* A4, 15834 and 8196 strains, using a sterile needle. Incubations were performed at 22 ± 2 °C under a 12 h photo-period.

Within two to four weeks after the infection, genetically different hairy root clones (HRC) appeared on the site of injury. To eliminate the bacteria, the clones were sub-cultured to solid MS media (+ 3.0% (*w*/*v*) sucrose, solidified with 0.7% agar) supplemented with 1 g/L ampicillin (Sigma) and 250 mg/L cefotaxime (Sigma) two times for four weeks, then with the addition of 500 mg/L ampicillin (Sigma, Budapest, Hungary) and 125 mg/L cefotaxime (Sigma) two times for four weeks. After the disinfection period, media with 2% sucrose were used. The HRCs were grown in darkness at 22 ± 2 °C. After two subcultures onto solid MS media (without antibiotics), HRCs were transferred to 100 mL Erlenmeyer flasks containing 40 mL liquid MS media (without agar). They were subsequently cultivated on a gyratory shaker at 100 rpm under the same conditions as those described above. Tips of the growing HRCs were sub-cultured every four weeks.

### 4.2. Confirmation of Transformation by PCR

Transformation was confirmed by PCR after DNA isolation (Promega Kit, Madison, WI, USA) from all of the clones, from the mother plant as a negative control and from *A. rhizogenes* A4 bacteria as a positive control. For the PCR reaction, *RolC* primers (synthetized by Bio Basic Canada Inc. [35]) and Promega Kit were used. We used a modified PCR program of Soudek et al. [35]: denaturation 2 min at 94 °C; amplification cycle for 30 times: 0.5 min at 94 °C, 1 min annealing at 59 °C, 1.5 min DNA synthesis at 72 °C; then samples were kept at 4 °C in the Bio-Rad iCycler machine (Hercules, CA, USA). After agarose gel electrophoresis (55 V) *rolC* genes were detected by UV light at 260 nm. Two technical replicates were run, showing identical results.

### 4.3. Biomass Production

HRCs were cultured in 100 mL Erlenmeyer flasks containing 40 mL liquid MS media. They were cultivated on a gyratory shaker at 100 rpm under the same conditions as those described above. Before transferring the HRC tips, flasks containing the media were weighed on an analytical scale. After sub-culturing the HRC tips in laminar air flow hood, flasks containing both media and HRC tips were scaled again. Then the weight of transferred HRCs was calculated. After four weeks of cultivation and laying of medium HRC samples fresh weight were scaled. Biomass production was expressed as a daily growth index (DGI = (final weight/starting weight)/days of culture [74]). To calculate dry material content (%), dry weight was also determined from lyophilized samples.

### 4.4. Gas Chromatography Measurements

Sample preparation: fresh, four-week-old culture samples were analyses by GC-MS. Accurately weighed, approximately 350 mg of fresh samples were crushed with quartz sand in Eppendorf tubes, and incubated for 7 min. Thereafter, 700 µL acetone was added to the samples. Samples were vortexed and centrifuged for 1 min at 13,000 rpm. The supernatant was centrifuged again under the same conditions, and filtered through a 0.22 µm RC membrane filter (FilterBio RC Syringe Filter; Labex Ltd., Budapest, Hungary) for analysis. Results are presented on a dry weight basis.

GC-MS: The method was a modified method described in Szűcs et al. [5]. Injection was carried out in split mode (15:1 split ratio), injection volume was 2 µL. The GC-MS analysis was performed on an Agilent 6890 GC, equipped with a 5973N mass selective detector, and Chrom Card Server Ver.1.2. software (Santa Clara, CA, USA). Capillary column: 30 m × 0.25 mm × 0.25 μm, SLB-5 ms 5% phenyl-methyl syloxane. Carrier gas: He. Rate of flow: 1.6 mL/min. Temperature program: 50 °C (3 min); by 15 °C/min to 200 °C (2 min); by 40 °C/min to 280 °C (1 min). Analysis: 18 min. MS conditions: 70 eV ionisation energy, 40–500 *m*/*z* mass range (scan mode). Peak identification: based on standards, retention times, comparing the mass spectra in NIST 05 library and comparing the data to that in the literature. The major components (PEITC, MeSPITC) were integrated from TIC (total ion chromatogram). The integration of minor components was made in SIM (selected ion monitoring) mode using the following characteristic m/z product ions: AITC (99, 72, 41), PECN (91, 65, 43), I3ACN (156, 155, 130, 101) (Table S2). Four-point calibration curves were made from AITC (Sigma) and PEITC (Sigma), in the 0.00976 µg/mL to 1.25 µg/mL range.

### 4.5. Liquid Chromatography Measurements

Sample preparation: water extracts from four-week-old lyophilized samples were analyzed by LC-ESI-MS/MS. Accurately weighed ~50 mg DW samples were heated at 80 °C for 5 min to inactivate the myrosinase enzyme. After homogenization with quartz sand in Eppendorf tubes, 1000 µL of boiling sterile bi-distilled water was added. The samples were vortexed, then heated in a water bath at 90 °C for 10 min. The vortexed samples were centrifuged at 3000 rpm for 10 min. The supernatant was centrifuged again under the same conditions, then the supernatant from the second centrifugation cycle was diluted to 5 mL with sterile bidistilled water, then a 1.5 mL aliquot was filtered through a 0.22 µm RC membrane filter (FilterBio RC Syringe Filter; Labex Ltd.) for analysis.

RP-HPLC: an Agilent 1100 HPLC system (Santa Clara, CA, USA)was used; column: Zorbax SB-C18 (150 × 3.0 mm; I.d. 3.5 µm), maintained at 30 °C; eluents: A: 0.1% formic acid, B: methanol; gradient: 0–30 min from 10% to 40% B, 30–31 min from 40% to 100% B, 31–37 min 10% B, flow rate: 0.3 mL/min; injection volume: 5 µL.

RP-HPLC: an Agilent 1100 HPLC system was used; column: Zorbax SB-C18 (150 × 3.0 mm; i.d. 3.5 µm), maintained at 30 °C; eluents: A: 0.1% formic acid, B: methanol; gradient: 0–30 min from 10% to 40% B, 30–31 min from 40% to 100% B, 31–37 min 100% B, 37–38 min from 100% to 10% B, flow rate: 0.3 mL/min; injection volume: 5 µL.

LC-ESI-MS/MS: an Agilent 6410 Triple Quadrupole Electrospray ion source was used in negative ion mode. ESI settings: nitrogen gas temperature 350 °C, nebulizer pressure 45 psi, drying gas flow rate 9 L min^−1^, capillary voltage: 3500 V, fragmentor voltage: 100 V; Collision energy: 25–30 eV (depending on the structure). Data about collision energies, precursor and product ions can be seen in Table S1. Q2 fragments of identified GLSs are presented in Figure S2.

The used method was based on Bennett et al. [45], Argentieri et al. [46], Agneta et al. [50] and Sansom et al. [47].

### 4.6. Myrosinase Activity Analysis

Sample preparation and gel electrophoresis was undertaken by the method of Gonda et al. [15].

Sample preparation: Fresh four-week-old samples were grinded with silica sand in Eppendorf tubes, than 20 mM pH 6.5 sodium-phosphate buffer was added to the samples in 1:1 rate in fresh rate basis. Samples were vortexed and centrifuged for 10 min at 13,000 rpm at 4 °C. From the supernatant protein contents of the sample extracts were measured by Bradford probe. Enzyme assays were referred to protein units. 

Gel electrophoresis: On the native polyacrylamide gels (5.7% top/stacking and 10 or 7.5% bottom/resolving gels) 100 µg protein/µL samples were analysed, completed with ×5 cracking. Four gels were electrophoresed at the same time at 50 mA for 3 h at 4 °C in dark. Gels were washed two times with distillated water, then two times with 1 mM sodium-phosphate buffer until the pH level decreased to 7.3. 

Detection was made by a solution, containing 1 mM sodium-phosphate (5% of the whole solution), 1 mM ascorbic acid (1%), 0.01% methylred indicator (10%), 2.5 mg/mL sinigrin (25%) and 59% distillated water. Evaluation was made by CP Atlas 2.0 gel image processing software (green channel).

### 4.7. Peroxidase Content and Activity

Sample preparation: samples were homogenized with quartz sand in Eppendorf tubes after harvest at the end of the culture perios (4 weeks). Then, 50 mM pH 7.5 sodium-phosphate buffer was added to the samples at 1:1 ratio (mg:µL). Samples were vortexed and centrifuged for 10 min at 13,000 rpm at 4 °C. The protein contents of the supernatants were measured by Bradford assay (Bradford 1976). Enzyme assays were expressed as activity per protein units (mmol pyrogallol min^−1^ mg^−1^ protein, mmol guaiacol min^−1^ mg^−1^ protein). The same method of gel electrophoresis and spectrophotometry was used like in the article of M.Hamvas et al. [75].

### 4.8. Statistical Analysis

Statistical analyses were carried out in R 3.5.2. [76] and were visualized using the package ggplot2 [77].

ChemAxon MarvinSketch was used for drawing chemical structures and reactions [78].

## 5. Conclusions

In the present study, we would like to outline that in case of HRC induction, the variability of clones could be increased through different plant organ inoculation with agrobacteria. Although the most wide-spread target organ is the leaf blade, other plant organs e.g., the petiole can be treated to create HRCs, yet, with different metabolite and enzyme patterns. The expression of enzymes and other proteins are usually organ-specific in native plants. This likely translates into the organ-specific presence of MYR isoenzymes in HRCs, which contributes to different patterns and concentrations of the biologically active compound content of horseradish HRCs, as shown by organ-dependent differences between many members of the GLS–MYR–ITC/nitrile system.

## Figures and Tables

**Table 1:** GLSs and their respective ITC and nitrile products [5,51,52,53].NA, not available.

GLS	GLS Breakdown Products	
	ITC	Nitrile [43,44]
Sinigrin (SIN) ^a^	Allyl ITC (AITC) ^a^	allyl cyanide
Glucoiberverin (IBER) ^a^	3-(methylthio)propyl ITC (MeSPITC) ^a^	4-(methylthio)-butanenitrile
Glucoibarin (GIB) ^a^	7-(methylsulfinyl)heptyl ITC	NA
Glucobrassicin (BRASS) ^a^	3-indolylmethyl ITC	indol-3-acetonitrile (I3ACN) ^a^
Gluconasturtiin (GLN) ^a^	2-phenylethyl ITC (PEITC) ^a^	3-phenylpropionitrile (PECN) ^a^
4-methoxy -or neoglucobrassicin (NEO) ^a^	4-methoxyindol-3-ylmethylor 1-methoxyindol-3-ylmethyl ITC	1-methoxyindol-3-acetonitrile
Glucoarabishirsutain (ARAB) ^a^	7-(methylthio)-heptyl ITC	NA

^a^ Compounds, which were detected from *A. rusticana* hairy root cultures in this study.

**Table 2:** Statistical difference between the two horseradish hairy root clone groups distributed based on the inoculated organ (petiole or leaf blade), for the different examined features (variables).

Variable	*p* Value	Significance
Branching	0.06823	
AdShoots	0.43899	
PinkExtract	0.05616	
DWpc	0.83269	
DGI	0.04864	*
MyrB1	0.76511	
MyrB2	0.08308	
MyrB3	0.66652	
SIN	0.00730	**
IBER	0.03864	*
GIB	0.00277	**
BRASS	0.00291	**
GLN	0.09426	
NEO	0.00153	**
ARAB	0.03068	*
AITC	0.10321	
PECN	0.00072	***
MeSPITC	0.34163	
PEITC	0.48132	
I3ACN	0.00025	***
ProtContent	0.94386	
HRP_pg	0.62207	
HRP_gu	0.23127	
HRP_pg_AUC	0.01124	*
HRP_gu_AUC	0.00745	**

Statistical significance: *t*-test, two-sided, *** *p* < 0.001, ** *p* < 0.01, * *p* < 0.05. Abbreviations: Branching—branching of the HRCs (visual evaluaion); AdShoots—adventitious shoot formation of the HRCs (visual evaluation); PinkExtract—pink color of water extracts for GLS measurement (visual evaluation); DWpc—dry weight %; DGI—daily growth index; MyrB1, 2, 3—1., 2., 3. activity values of myrosinase bands, on-gel detection; the rest of the abbreviations are the same as in Figure 3.

## Supplementary Materials

The following are available online at https://www.mdpi.com/1420-3049/24/15/2828/s1, **Figure S1:** PCR analysis of *Armoracia rusticana* hairy root cultures, transformed by *Agrobacterium rhizogenes* A4, with positive (DNA isolated from *Agrobacterium rhizogenes* A4, C+), negative control (DNA isolated from mother plant leaf), blank (no DNA added) samples., **Figure S2:** Q2 fragments of identified and putatively identified GLSs in *Armoracia rusticana* hairy root cultures. (**a**) Sinigrin: 75.3, 96.9, 195.1, 358.0; (**b**) glucoiberverin: 74.9, 79.0, 259.1, 274.7, 405,0; (**c**) glucoibarin: 97, 414,0, 478.0; (**d**) glucobrassicin: 75.0, 96.8, 162.6, 205.2, 258.7; (**e**) gluconasturtiin: 75.2, 97.0, 180.2, 229.0, 259.0, 422.0; (**f**) 4-methoxy- or neoglucobrassicin: 97.0, 195.0, 477.0; (**g**) glucaorabishirsutain: 74.8, 97.0, 274.9, 462.1. Identification was based on literature data [48,49] and comparison with authentic standards (GLN and SIN). **Figure S3:** Representative BPC scan chromatogram of *Armoracia rustica* HRCs transformed by *Agrobacterium rhizogenes* A4, by LC-MS/MS (fragmentor voltage 100 V). Identified and putatively identified natural GLSs: 1: sinigrin; 2: glucoiberverin, 3; glucoibarin; 4: glucobrassicin; 5: gluconasturtiin; 6: 4-methoxy- or neoglucobrassicin; 7: glucaorabishirsutain. Identification was based on Agneta et al. [2,51] and comparison with authentic standards (GLN and SIN)., **Figure S4**: Representative XIC chromatogram of *Armoracia rustica* HRCs transformed by *Agrobacterium rhizogenes* A4, by GC-MS. Identified and putatively identified natural GLS breakdown products: 1: AITC; 2: PECN, 3; MeSPITC; 4: PEITC; 5: I3ACN. Identification was based on NIST 05 library, and on literature data [5,13,47,55], as well as comparison with authentic standards PEITC and AITC., **Figure S5:** Horseradish peroxidase analysis of *Armoracia rusticana* hairy root cultures by on-gel detection, transformed by *Agrobacterium rhizogenes* A4. Samples include horseradish root, in vitro horseradish plantlet leaves, hairy root clones of leaf blade or petiole origin (ArLB, and ArP, respectively) and with five unit HRP enzyme standard (St5U)., **Figure S6:** Visually evaluated morphological differences among *Armoracia rusticana* hairy root culture lines, transformed by *Agrobacterium rhizogenes* A4. (**a**)–(**c**): Adventitious shoot formation; values: (**a**): 0; (**b**): 1; (**c**): 2. (**d**)–(**f**): Branching; values: (**d**): 0; (**e**): 1; (**f**): 2. **Figure S7:** (**a**) Correlation heatmap and hierarchical clustering of features of horseradish hairy root culture lines, from petiole origin. (**b**) Correlation heatmap and hierarchical clustering of features of horseradish hairy root culture lines, from leaf blade origin. Compounds are sorted according to their order obtained by hierarchical clustering analysis of the Minkowski distance matrix of the scaled and centered feature dataset. Clustering was accomplished by Ward’s method. Color is proportional to Pearson’s correlation value between two features. **Figure S8:** Correlation between myrosinase isoenzyme activity and GLS breakdown products in hairy root lines of *Armoracia rusticana*. Axis labels: MeSPITC, 3-(methylthio)propyl isothiocyanate (abundance measured by GC-MS); MyrB2, activity of a myrosinase isoenzyme, as measured by on-gel evaluation; PECN - 3-phenylpropionitrile (abundance measured by GC-MS). **Table S1**: Identified and putatively identified natural GLSs from *Armoracia rustica* HRCs transformed by *Agrobacterium rhizogenes* A4, by LC-MS/MS (fragmentor voltage 100 V). Identification was based on Agneta et al. [2,51] and comparison with authentic standards (GLN and SIN). **Table S2**: Identified and putatively identified natural ITCs and nitriles from *Armoracia rustica* HRCs transformed by *Agrobacterium rhizogenes* A4, measured by GC-MS. Identification was based on NIST 05 library, and on literature data [5,13,47,55], as well as comparison with authentic standards PEITC and AITC. Table S3: Raw dataset of measured features of the *Armoracia rusticana* hairy root cultures.

## Abbreviations

AdShoots—adventitious shoots; AITC—allyl isothiocyanate; ARAB—glucoarabishirsutain; ArLB—*Armoracia rusticana* hairy root clones initiated from leaf blade; ArP—*Armoracia rusticana* hairy root clones initiated from petiole; Branching—branching of hairy root cultures; BRASS—glucobrassicin; CYP—cytochrome P enzyme family; DNA—deoxyribonucleic acid; DWpc—dry weight %; GC-MS—gas chromatography-mass spectrometry; GIB—glucoibarin; GLN –gluconasturtiin; GLS—glucosinolate; GST—glutathion-S-transferase; DGI—daily growth index; HRC—hairy root clone; HRP—horseradish peroxidase; IAA—indole acetic acid; IBER—glucoiberverin; ITC—isothiocyanate; I3ACN—indole-3-acetonitrile; LC-ESI-MS/MS—liquid chromatography electronspray ionisation-mass spectrometry; MYR—myrosinase; MyrB1, 2, 3—different myrosinase bands on gel detection; MeSPITC—3-methyl tiopropyl isothiocyanate; NEO—neoglucobrassicin; PECN—3-phenylpropionitrile; PEITC—2-phenylethyl isothiocyanate; PCR—Polymerase chain reaction; RNA—Ribonucleic acid.
