# Supplementary material for: Variability of Bioactive Glucosinolates, Isothiocyanates and Enzyme Patterns in Horseradish Hairy Root Cultures Initiated from Different Organs
Source: Molecules. 2019 Aug 2;24(15):2828. doi: 10.3390/molecules24152828 (PMC6696319; doi:10.3390/molecules24152828)
Supplement: Supplementary file 1 [file molecules-24-02828-s001.zip › supplementary.v6.pdf]

Variability of Bioactive Glucosinolates, Isothiocyanates and Enzyme Patterns in Horseradish Hairy Root Cultures Initiated from Different Organs

Regina Bertóti [1,2], Andrea Böszörményi [1], Ágnes Alberti [1], Szabolcs Béni [1], Márta M-Hamvas [2], Éva Szőke [1], Gábor Vasas [2] and Sándor Gonda [2,*]

[1] Department of Pharmacognosy, Semmelweis University, Üllői út 26, H-1085 Budapest, Hungary
[2] Department of Botany, Division of Pharmacognosy, University of Debrecen, Egyetem tér 1, H-4010 Debrecen, Hungary

Supplementary Material

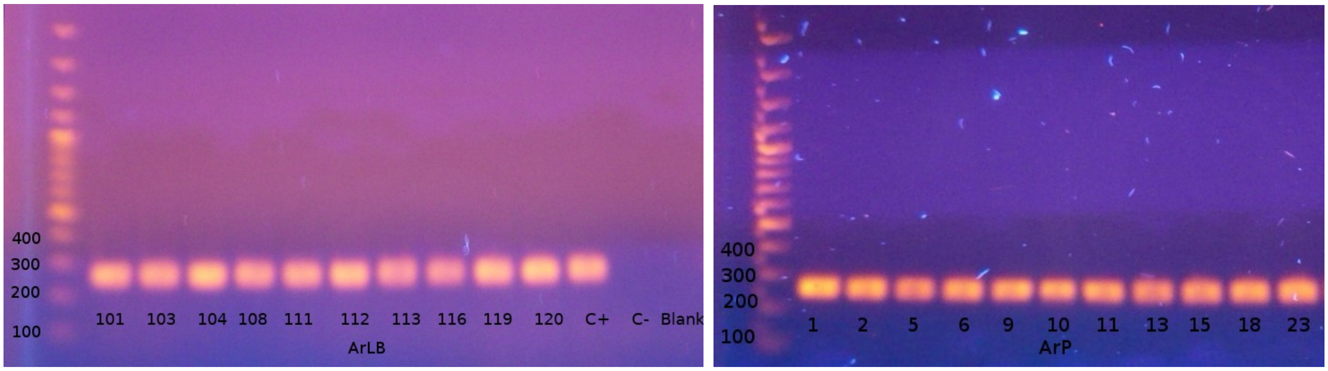

Figure S1: PCR analysis of *Armoracia rusticana* hairy root cultures, transformed by *Agrobacterium rhizogenes* A4, positive control (DNA isolated from *Agrobacterium rhizogenes* A4, C+), negative control (DNA isolated from mother plant leaf), blank (no DNA added) samples.

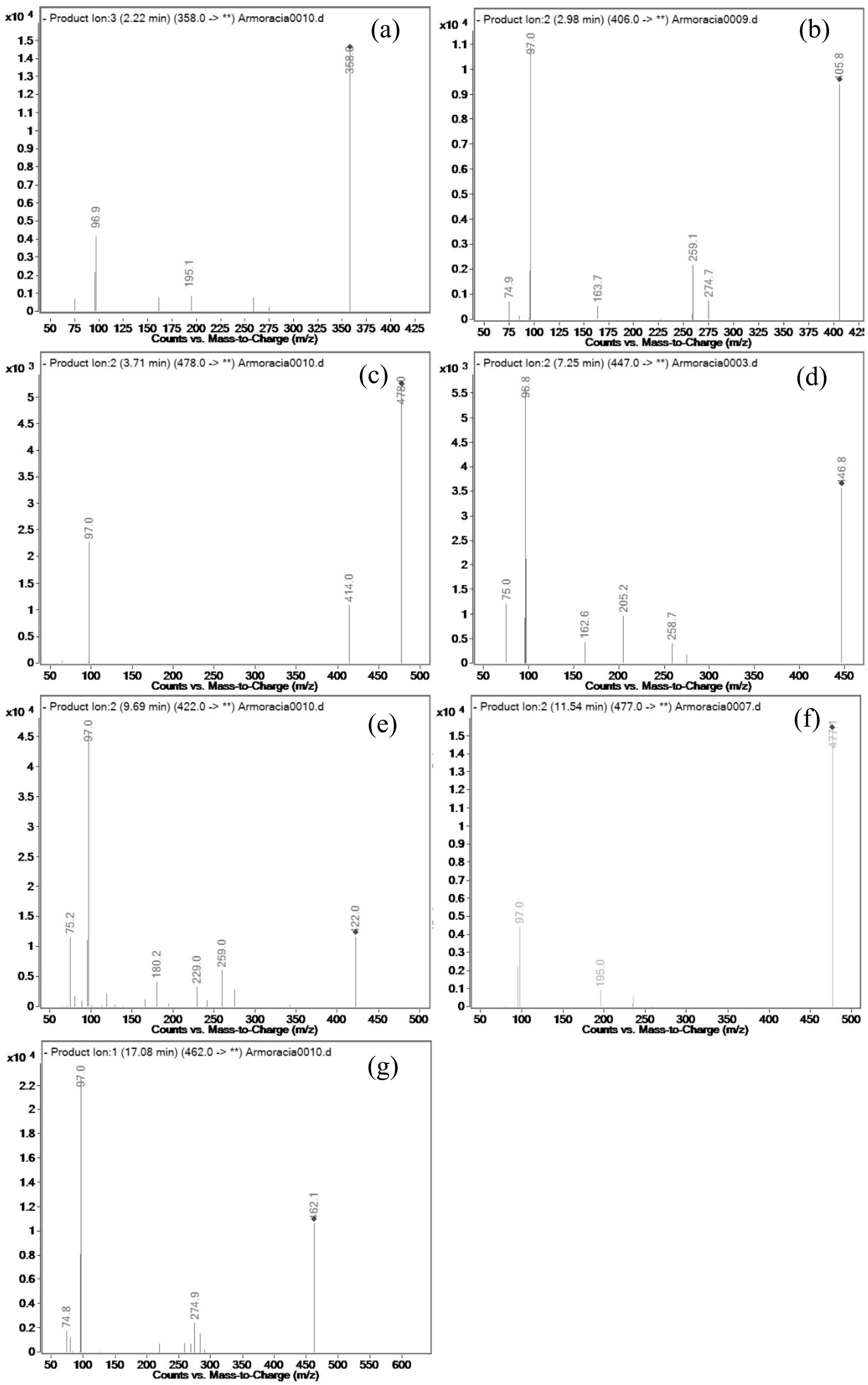

Figure S2: Major MS^2 fragments of identified and putatively identified GLSs in *Armoracia rusticana* hairy root cultures. (a) Sinigrin: 195.1, 96.9, 75.3; (b) glucoiberverin: 274.7, 259.1, 79.0, 74.9; (c) glucoibarin: 414,0, 97; (d) glucobrassicin: 258.7, 205.2, 162.6, 96.8, 75.0; (e) gluconasturtiin: 259.0, 229.0, 180.2, 97.0, 75.2; (f) 4-methoxy- or neoglucobrassicin: 195.0, 97.0; (g) glucaorabishirsutain: 274.9, 97.0, 74.8. Identification was based on literature data [1–3] and comparison with authentic standards (GLN and SIN). Note the characteristic GLS fragment (sulfoglucose) 259 and fragment 75 of H_3SCN were found in all putatively idenified GLS molecules, at least as minor fragment. Fragment 97 of HSO_4 was major fragments in all examined GLS molecules.

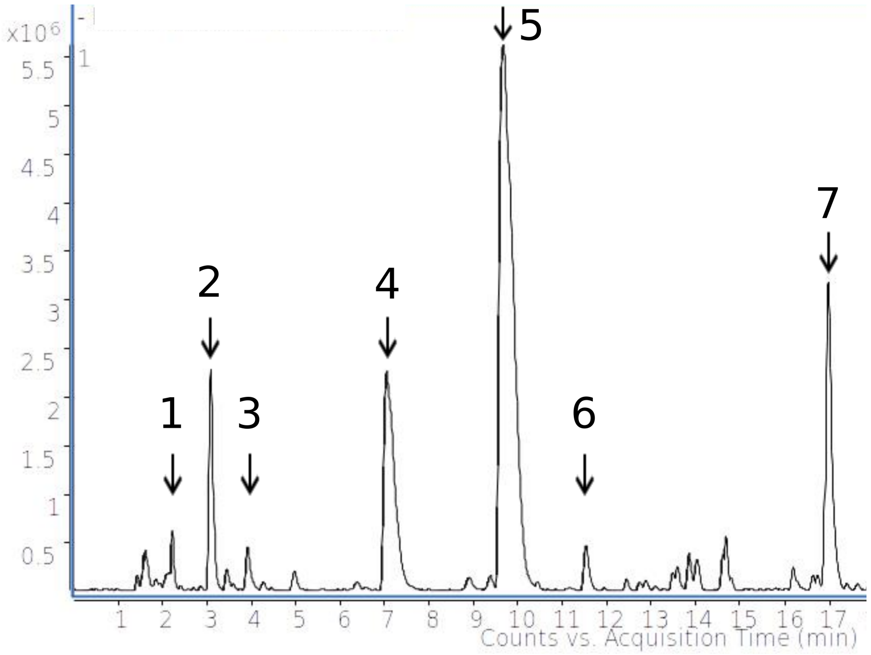

Figure S3: Representative BPC scan chromatogramm of *Armoracia rustica* HRCs transformed by *Agrobacterium rhizogenes* A4, by LC-MS/MS (fragmentor voltage 100 V). Identified and putatively identified natural GLSs: 1: sinigrin; 2: glucoiberverin, 3; glucoibarin; 4: glucobrassicin; 5: gluconasturtiin; 6: 4-methoxy-glucobrassicin, or neoglucobrassicin; 7: glucaorabishirsutain. Identification was based on Agneta et al [3,4] and comparison with authentic standards (GLN and SIN).

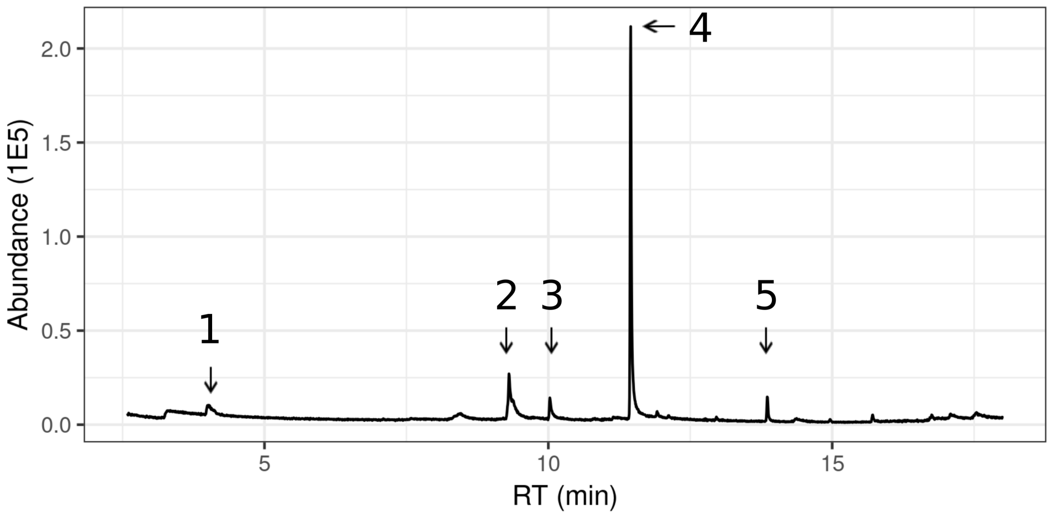

Figure S4: Representative XIC chromatogramm of *Armoracia rusticana* HRCs transformed by *Agrobacterium rhizogenes* A4, by GC-MS. Identified and putatively identified natural GLS breakdown products: 1: AITC; 2: PECN, 3; MeSPITC; 4: PEITC; 5: I3ACN. Identification was based on NIST 05 library, and on literature data [5–8], as well as comparison with authentic standards PEITC and AITC.

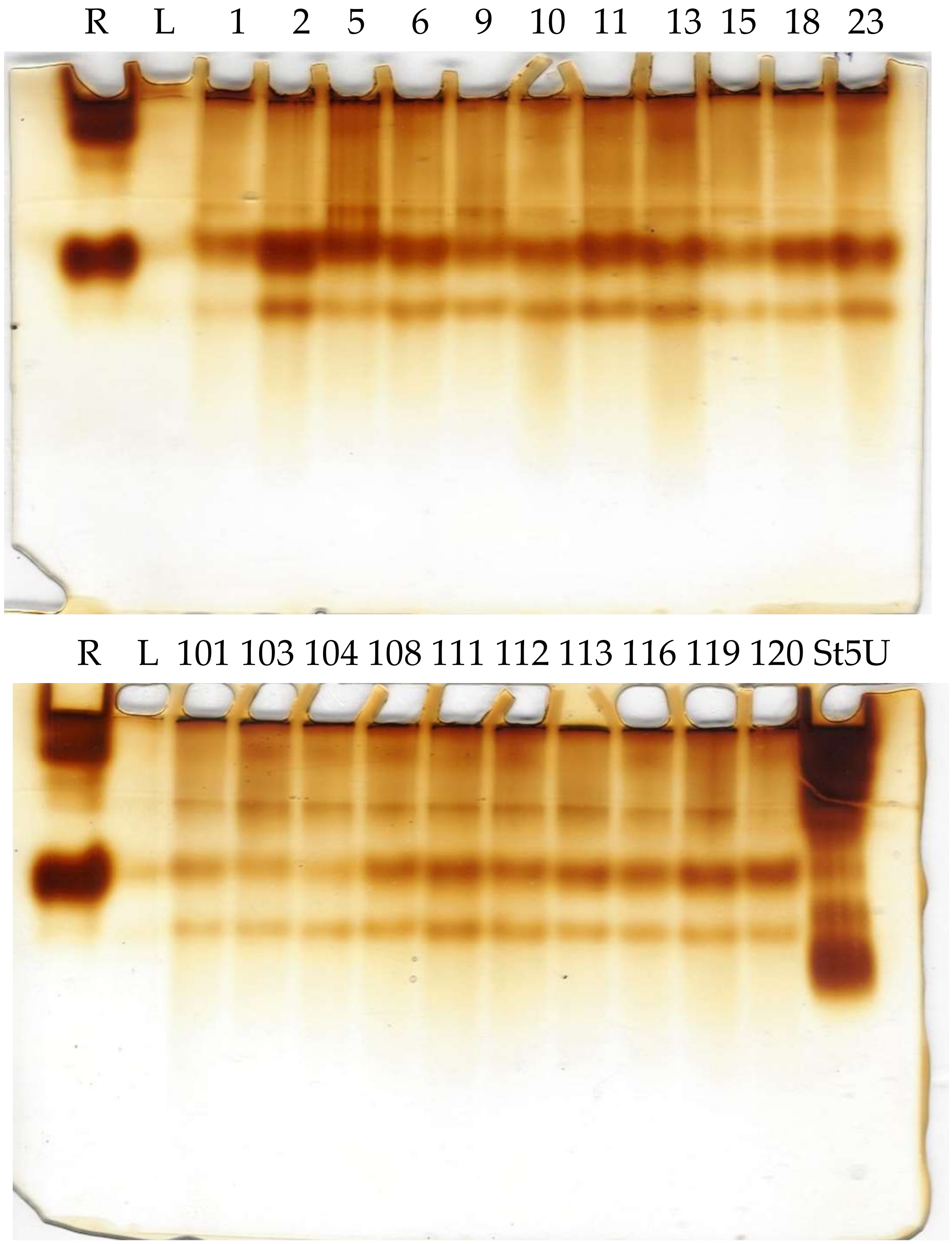

Figure S5. Horseradish peroxidase analysis of *Armoracia rusticana* hairy root cultures by on-gel detection, transformed by *Agrobacterium rhizogenes* A4. Samples include: R, horseradish root, L, *in vitro* horseradish plantlet leaves, hairy root clones of leaf blade ("ArLB", 101-120) or petiole origin ("ArP", 1-23) and with 5 unit HRP enzyme standard (St5U).

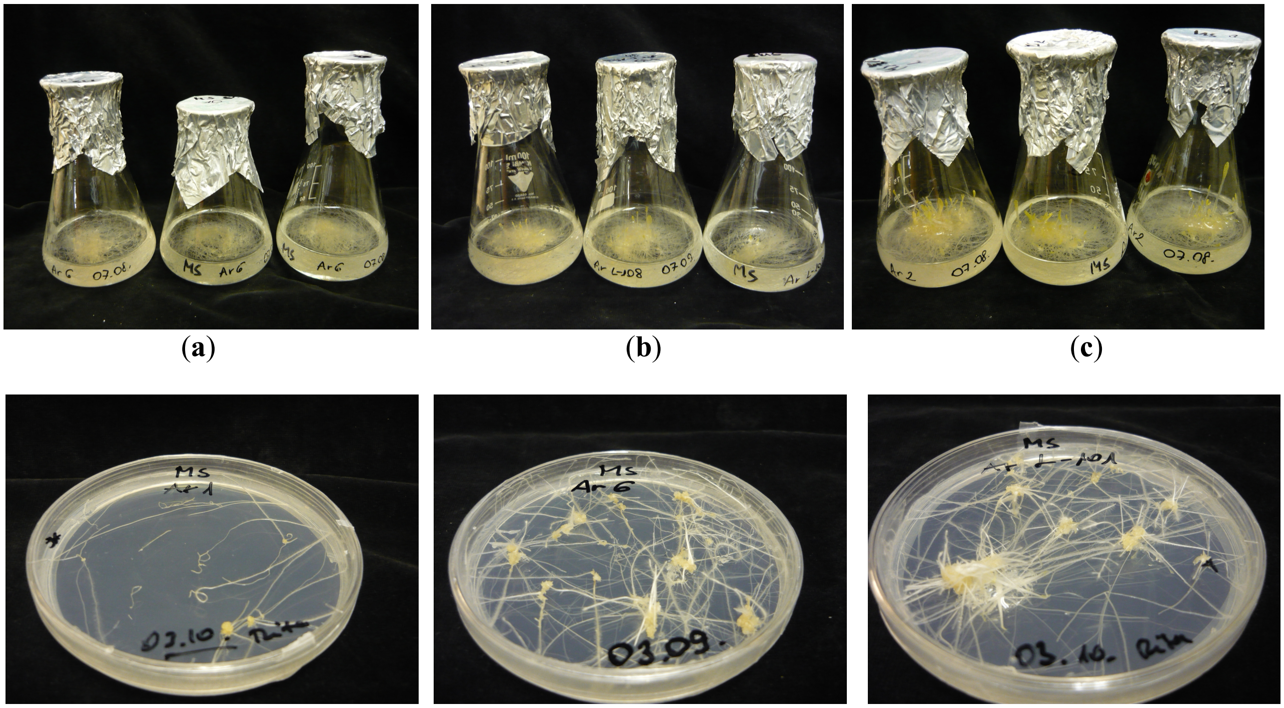

(**a**) (**b**) (**c**)

(**d**) (**e**) (**f**)

Figure S6: Visually evaluated morphological differences among *Armoracia rusticana* hairy root culture lines, transformed by *Agrobacterium rhizogenes* A4. **(a)-(c)**: Adventitious shoot formation; values: (a): 0; (b): 1; (c): 2. **(d)-(f)**: Branching; values: (d): 0; (e): 1; (f): 2.

(a)

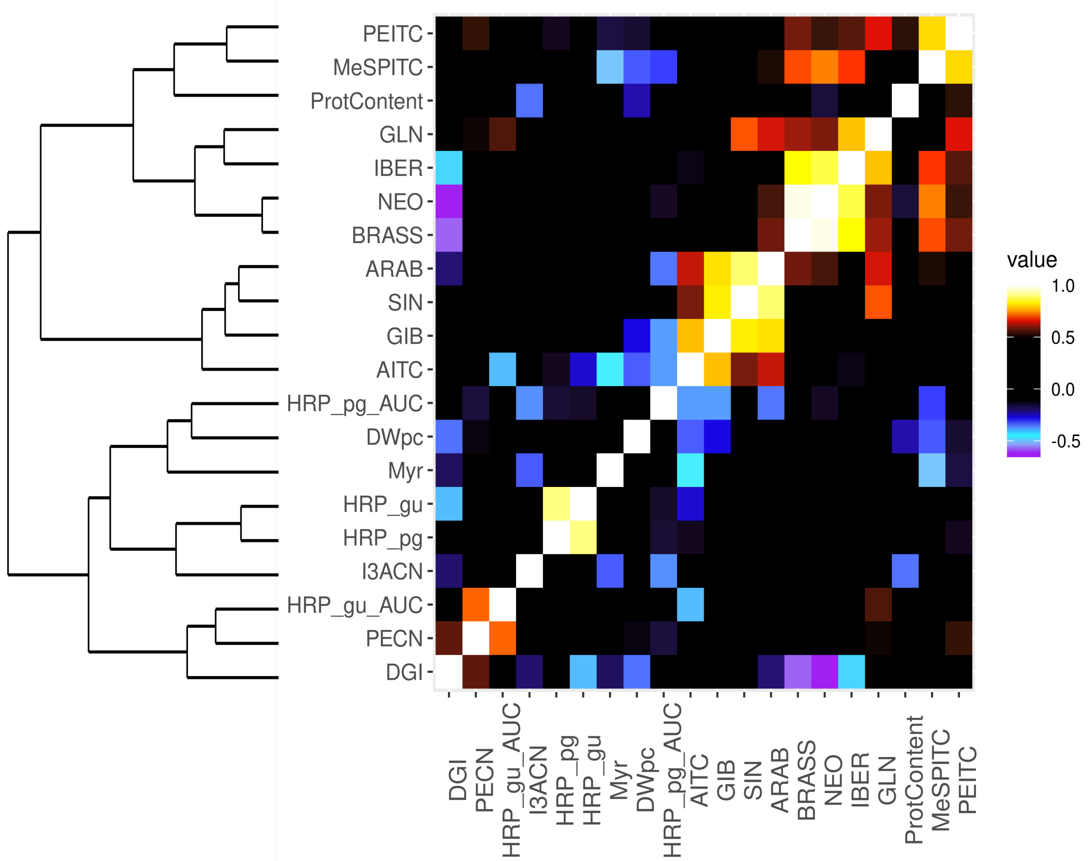

(b)

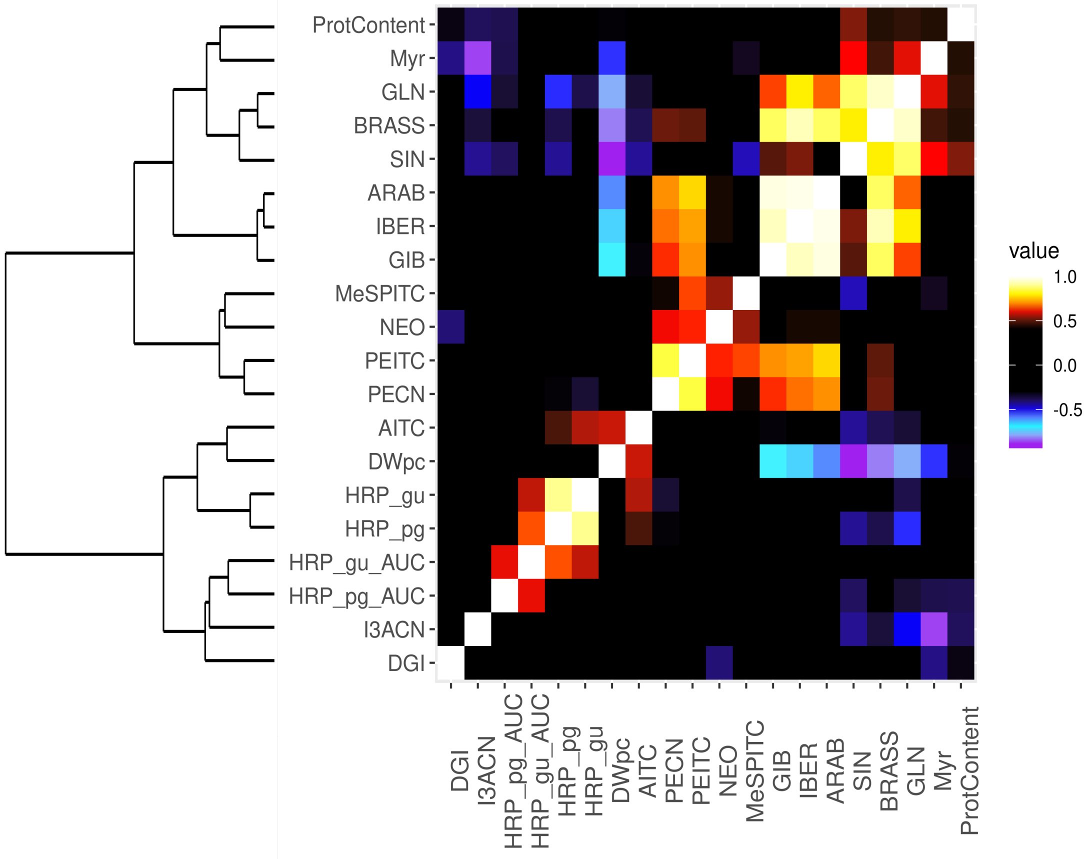

Figure S7: (a) Correlation heatmap and hierarchial clustering of features of horseradish hairy root culture lines, from petiole origin. **(b)** Correlation heatmap and hierarchial clustering of features of horseradish hairy root culture lines, from

leaf blade origin. Compounds are sorted according to their order obtained by hierarchical clustering analysis of the Minkowski distance matrix of the scaled and centered feature dataset. Clustering was accomplished by Ward's method. Color is proportional to Pearson's correlation value between two features. Feature abbreviations: PECN - 3-phenylpropionitrile (abundance); HRP_gu_AUC - horseradish peroxidase content with guaiacol as substrate (abundance); DGI – daily growth index; ProtContent - protein content (mg protein mg fresh weight^{-1}); I3ACN – indole-3-acetonitrile (abundance); HRP_pg – horseradish peroxidase activity with pyrogallol as substrate (mmol pyrogallol min^{-1} mg^{-1} protein); HRP_gu - horseradish peroxidase activity with guaiacol as substrate (mmol guaiacol min-1 mg^{-1} protein); HRP_pg_AUC - horseradish peroxidase content with pyrogallol as substrate (abundance); Myr – myrosinase activity; DWpc – dry weight %; AITC – allyl isothiocyanate (µg mg^{-1} DW); GIB – glucoibarin (abundance); SIN – sinigrin (µg mg^{-1} DW); ARAB – glucoarabishirsutain (abundance); MeSPITC – 3-(methylthio)propyl isothiocyanate (abundance); PEITC – 2-phenylethyl isothiocyanate (µg mg^{-1} DW); BRASS – glucobrassicin (abundance); NEO – neoglucobrassicin (abundance); IBER – glucoiberverin (abundance); GLN – gluconasturtiin (µg mg^{-1} DW).

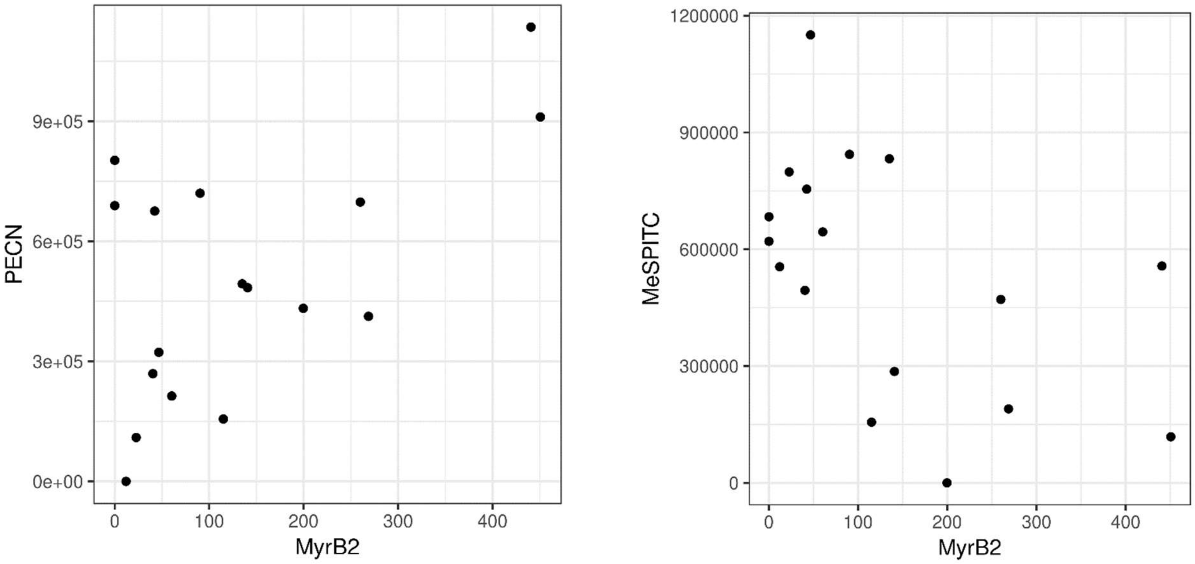

(a) (b)

Figure S8. Correlation between myrosinase isoenzyme activity and GLS breakdown products in hairy root lines of *Armoracia rusticana*. Axis labels: MeSPITC, 3-methyltiopropyl isothiocyanate (abundance measured by GC-MS); MyrB2, activity of a myrosinase isoenzyme, as measured by on-gel evaluation; PECN - 3-phenyl-propio-nitrile (abundance measured by GC-MS).

Table S1. Identified and putatively identified natural GLSs from *Armoracia rustica* HRCs transformed by *Agrobacterium rhizogenes* A4, by LC-ESI-MS/MS (fragmentor voltage 100 V). Identification was based on Agneta et al [3,4], Rochfort et al [2] and Fabre et al [1], and comparison with authentic standards (GLN and SIN). MS^2 freagments, which were used for quantitative analysis are presented.

ID	(Putative) GLS identification	$[M-H]^-$	Rt (min) [1]	GLS class	MS^2	CE (V) [2]
1	Sinigrin (SIN)	358	2.22	Aliphatic	195.1; 97.0; 75.3	30
2	Glucoiberverin (IBER)	406	2.98	Aliphatic	258.8; 97.0; 75.1	30
3	Glucoibarin (GIB)	478	3.71	Aliphatic	414.0; 97.0	25
4	Glucobrassicin (BRASS)	447	7.06	Indol	258.8; 205.0; 96.9; 75.0	25
5	Gluconasturtiin (GLN)	422	9.69	Aromatic	259.0; 180.2; 97.0; 75.2	25
6	Neoglucobrassicin (NEO)	477	11.63	Indol	259.1;195.2; 74.7; 96.8	25
7	Glucoarabishirsutain (ARAB)	462	17.08	Aliphatic	274.9; 97.0; 74.8	30

[1] retentiontime; [2] collision energy

Table S2. Identified and putatively identified natural ITCs and nitriles from *Armoracia rustica* HRCs transformed by *Agrobacterium rhizogenes* A4, measured by GC-MS. Identification was based on NIST 05 library, and on literature data [5–8], as well as comparison with authentic standards PEITC and AITC.

ID	(Putative) ITC/nitrile identification	$[M-H]^-$	Rt (min) [1]	ITC/nitrile class	MS^2	Precursore GLS
1	Allyl ITC (AITC)	99	4.6	Aliphatic	99; 72; 41	Sinigrin (SIN)
2	3-phenylpropionitrile (PECN)	131	9.3	Aliphatic	91; 65; 43	Gluconasturtiin (GLN)
3	3-(methylthio)propyl ITC (MeSPITC)	147	10.0	Aliphatic	101; 72; 61; 41	Glucoiberverin (IBER)
4	2-phenylethyl ITC (PEITC)	163	11.4	Aromatic	163;105; 91	Gluconasturtiin (GLN)

[1] retention time

Table S3: Raw dataset of measured features of the *Armoracia rusticana* hairy root cultures. Please see the .CSV file for the values.

Compound name abbreviations: AITC – allyl isothiocyanate; ARAB – glucoarabishirsutain; BRASS – glucobrassicin; GIB – glucoibarin; GLN – gluconasturtiin; I3ACN – indole-3-acetonitrile; IBER – glucoiberverin; MeSPITC – 3-(methylthio)propyl isothiocyanate; NEO – neoglucobrassicin; PECN - 3-phenylpropionitrile; PEITC – 2-phenylethyl isothiocyanate; SIN – sinigrin.

References

1. Fabre, N.; Poinsot, V.; Debrauwer, L.; Vigor, C.; Tulliez, J.; Fourasté, I.; Moulis, C. Characterisation of glucosinolates using electrospray ion trap and electrospray quadrupole time-of-flight mass spectrometry. Phyochem. Anal. 2007, 306–319.
2. Rochfort, S.J.; Trenerry, V.C.; Imsic, M.; Panozzo, J.; Jones, R. Class targeted metabolomics: ESI ion trap screening methods for glucosinolates based on MSn fragmentation. Phytochemistry 2008, 69, 1671–1679.
3. Agneta, R.; Rivelli, A.R.; Ventrella, E.; Lelario, F.; Sarli, G.; Bufo, S.A. Investigation of Glucosinolate Profile and Qualitative Aspects in Sprouts and Roots of Horseradish (*Armoracia rusticana*) Using LC-ESI–Hybrid Linear Ion Trap with Fourier Transform Ion Cyclotron Resonance Mass Spectrometry and Infrared Multiphoton Dissociation. *J. Agric. Food Chem.* **2012**, *60*, 7474–7482.55.
4. Agneta, R.; Möllers, C.; Rivelli, A.R. Horseradish (*Armoracia rusticana*), a neglected medical and condiment species with a relevant glucosinolate profile: a review. *Genet. Resour. Crop Evol.* **2013**, *60*, 1923–1943.
5. Szűcs, Z.; Plaszkó, T.; Cziáky, Z.; Kiss-Szikszai, A.; Emri, T.; Bertóti, R.; Sinka, L.T.; Vasas, G.; Gonda, S. Endophytic fungi from the roots of horseradish (*Armoracia rusticana*) and their interactions with the defensive metabolites of the glucosinolate - myrosinase - isothiocyanate system. *BMC Plant Biol.* **2018**, *18*.
6. Márton, M.-R.; Krumbein, A.; Platz, S.; Schreiner, M.; Rohn, S.; Rehmers, A.; Lavric, V.; Mersch-Sundermann, V.; Lamy, E. Determination of bioactive, free isothiocyanates from a glucosinolate-containing phytotherapeutic agent: A pilot study with in vitro models and human intervention. *Fitoterapia* **2013**, *85*, 25–34.
7. Sansom, C.E.; Jones, V.S.; Joyce, N.I.; Smallfield, B.M.; Perry, N.B.; van Klink, J.W. Flavor, Glucosinolates, and Isothiocyanates of Nau (Cook's Scurvy Grass, Lepidium oleraceum) and Other Rare New Zealand Lepidium Species. J. Agric. Food Chem. 2015, 63, 1833–1838.
8. Petrović, S.; Drobac, M.; Ušjak, L.; Filipović, V.; Milenković, M.; Niketić, M. Volatiles of roots of wild-growing and cultivated *Armoracia macrocarpa* and their antimicrobial activity, in comparison to horseradish, *A. rusticana. Ind. Crops Prod.* **2017**, *109*, 398–403.
